# Ketamine Reverses Lateral Habenula Neuronal Dysfunction and Behavioral Immobility in the Forced Swim Test Following Maternal Deprivation in Late Adolescent Rats

**DOI:** 10.3389/fnsyn.2018.00039

**Published:** 2018-10-30

**Authors:** Ryan D. Shepard, Ludovic D. Langlois, Caroline A. Browne, Aylar Berenji, Irwin Lucki, Fereshteh S. Nugent

**Affiliations:** Department of Pharmacology, F. Edward Hebert School of Medicine, Uniformed Services University of the Health Sciences, Bethesda, MD, United States

**Keywords:** lateral habenula, LHb, ketamine, intrinsic excitability, early life stress, maternal deprivation, depression, forced swim test

## Abstract

Mounting evidence suggests that the long-term effects of adverse early life stressors on vulnerability to drug addiction and mood disorders are related to dysfunction of brain monoaminergic signaling in reward circuits. Recently, there has been a growing interest in the lateral habenula (LHb) as LHb dysfunction is linked to the development of mental health disorders through monoaminergic dysregulation within brain reward/motivational circuits and may represent a critical target for novel anti-depressants, such as ketamine. Here, we show that maternal deprivation (MD), a severe early life stressor, increases LHb intrinsic excitability and LHb bursting activity, and is associated with the development of increased immobility in the forced swim test (FST) in late-adolescent male rats. A single *in vivo* injection of ketamine is sufficient to exert prolonged antidepressant effects through reversal of this early life stress-induced LHb neuronal dysfunction and the response in the FST. Our assessment of ketamine’s long-lasting beneficial effects on reversal of MD-associated changes in LHb neuronal function and behavior highlights the critical role of the LHb in pathophysiology of depression associated with severe early life stress and in response to novel fast-acting antidepressants.

## Introduction

Exposure to early-life stress is a strong predictor for later life mental disorders, including depression, suicide, post-traumatic stress disorder, schizophrenia and substance use disorder (Marco et al., 2015; Nemeroff, 2016). While the exact link between early-life stress and development of mental disorders is unknown, compelling evidence has accumulated implicating dysregulation of monoaminergic signaling in brain reward/motivational circuits as a culprit (Heim and Nemeroff, 2002; Rodrigues et al., 2011; Rentesi et al., 2013; Peña et al., 2017). Consistently, we found that a single 24 h episode of maternal deprivation (MD, a severe early life stressor as an established rodent model of child abuse and neglect) induces an epigenetic impairment of GABAergic synaptic plasticity within the ventral tegmental area (VTA), a midbrain area involved in reward-related processing, which could significantly contribute to dopamine (DA)-related reward dysregulation following this stress (Authement et al., 2015). Furthermore, our recent study suggests that MD-induced VTA DA neuronal dysfunction also involves a critical upstream brain area, the lateral habenula (LHb; Authement et al., 2018). Because of its rich reciprocal connectivity with forebrain limbic and midbrain structures, the LHb serves as a converging hub for cognitive and emotional signals that are conveyed to midbrain monoaminergic systems and, thus, plays a fundamental role in value-based decision-making and goal-directed behaviors. Not surprisingly, LHb dysfunction contributes to a myriad of cognitive, learning, emotional and social impairments associated with depression, anxiety, psychosis and drug addiction (Proulx et al., 2014; Graziane et al., 2018; Nuno-Perez et al., 2018).

Recently, ketamine, a non-competitive glutamatergic NMDA receptor (NMDAR) antagonist, has drawn interest due to its rapid and prolonged antidepressant actions after a single administration at low doses in patients suffering from treatment-resistant depression and in animal models of depression (Gerhard et al., 2016). Ketamine significantly reduces glucose utilization in the LHb of rats (Eintrei et al., 1999). Moreover, the antidepressant effects of ketamine in treatment-resistant major depression have been associated with decreased glucose utilization and metabolism in the habenula (Carlson et al., 2013) suggesting that antidepressant effects of ketamine may be related to reduction of LHb neuronal activity. Consistent with these findings, a recent study demonstrated that enhanced LHb neuronal bursting codes for behavioral depression and anhedonia in two rodent animal models of depression and that the rapid antidepressant effects of ketamine is mediated through local NMDAR-dependent blockade of this enhanced LHb neuronal bursting (Yang et al., 2018). Early MD also shifts the balance between synaptic excitation and inhibition towards excitation by increasing LHb intrinsic excitability while impairing responsiveness of LHb neurons to the stress hormone, corticotropin releasing factor (CRF) in juvenile rats (Authement et al., 2018). Similarly, chronic maternal separation triggers a depressive-like phenotype in adult mice that is associated with LHb hyperexcitability (Tchenio et al., 2017). Here, we extend our observations from juveniles to late adolescent rats where we found that MD-induced LHb hyperexcitability persists into late adolescence and is accompanied by an increase in LHb bursting activity and depressive-like behavior in the forced swim test (FST). Although many studies evaluating antidepressant-like effects of ketamine in animal models use depression models or acute stress paradigms in adults, here we used MD stress to explore the possibility that a single intraperitoneal (i.p.) injection of ketamine is sufficient to exert prolonged antidepressant effects through reversal of LHb neuronal dysfunction and behavioral despair induced by early life stress.

## Materials and Methods

All experiments were carried out in accordance with the National Institutes of Health (NIH) guidelines for the care and use of laboratory animals and were approved by the Uniformed Services University Institutional Animal Care and Use Committee. Data analysis was conducted in a blinded manner to ensure reproducibility.

### Maternal Deprivation and Slice Preparation

MD was carried out as previously described (Authement et al., 2015). Half of the male pups in litters of Sprague–Dawley rats (Taconic Farms) at P9 were isolated at 10:00 a.m. from the dam and their siblings for 24 h (MD group). The isolated rats were placed in a separate quiet room and kept on a heating pad (34°C) and not disturbed until being returned to their home cage 24 h later. The remaining non-separated male rat pups received the same amount of handling but were kept with the dam serving as the non-maternally deprived control group (non-MD group). Rats were maintained on a 12 h light/dark cycle and provided food and water ad libitum. The animals were taken for study during the light period, between 3 h and 5 h after the lights were turned on. Rats were weaned from the mother and housed in pairs in separate cages at P28. Separate cohorts of MD and non-MD (control) rats (age-matched) were used for behavioral experiments over P21–P28 (early-mid adolescent rats) or P42–P50 (late adolescent rats; McCormick and Mathews, 2010; Spear, 2016). Separate cohorts of late adolescent rats (P42–P50) were used for electrophysiological recordings in Figures 2C,D where one to three neurons were recorded per animal (*n* represents number of recorded cells/number of rats). On average four rat pups per litter contributed to the study (approximately a total of 42 litters). Rats were anesthetized with isoflurane and immediately decapitated. The brains were quickly dissected and placed into ice-cold artificial cerebrospinal fluid (ACSF) containing (in mM): 126 NaCl, 21.4 NaHCO_3_, 2.5 KCl, 1.2 NaH_2_PO_4_, 2.4 CaCl_2_, 1.00 MgSO_4_, 11.1 glucose, 0.4 ascorbic acid, saturated with 95% O_2_–5% CO_2_. Sagittal slices containing the LHb were cut at 250 μm and incubated in ACSF at 34°C for at least 1 h. Slices were then transferred to a recording chamber and perfused with ascorbic acid-free ACSF at 28°C.

**Figure 1:**
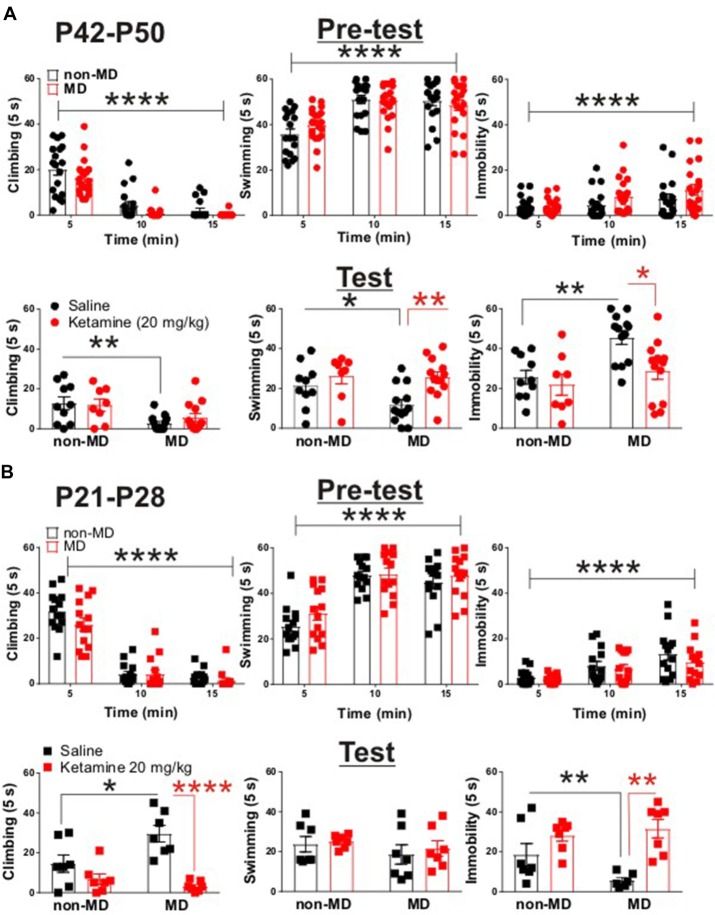
Ketamine normalized maternal deprivation (MD)-induced behavioral changes in forced swim test (FST) in adolescent rats. **(A)** Upper panel demonstrates the time course of behavioral transition from active (swimming and climbing) to passive (immobility) coping behaviors. Both non-MD and MD late-adolescent rats quickly learned to adopt immobility during the first swim session (Pre-test, non-MD: *n* = 18, MD: *n* = 26; climbing time course: *F*_(2,72)_ = 107.3, ****P < 0.0001; swimming time course: *F*_(2,72)_ = 39.72, ****P < 0.0001; immobility time course: *F*_(2,72)_ = 11.4, ****P < 0.0001; two-way ANOVA). Lower panel shows that MD induced a significant decrease in climbing behavior and a significant increase in immobility in FST (Test, non-MD + saline: *n* = 10, non-MD + Ketamine: *n* = 8, MD + saline: *n* = 13, MD + Ketamine: *n* = 13; swimming: *P < 0.05; climbing: **P < 0.01; immobility: **P < 0.01). Administration of ketamine (20 mg/kg) significantly decreased MD-induced immobility 24 h post-injection (swimming: *F*_(1,40)_ = 8.71, **P < 0.01; immobility: *F*_(1,40)_ = 6.27, *P < 0.01; two-way ANOVA). **(B)** Upper panel demonstrates the adoption of immobility in juvenile rats during Pre-test. Similar to late-adolescent rats, non-MD and MD juvenile rats quickly learned to adopt immobility during the first swim session (Pre-test, *n* = 14 in each group; climbing time course: *F*_(2,52)_ = 175.4, ****P < 0.0001; swimming time course: *F*_(2,52)_ = 82.48, ****P < 0.0001; immobility time course: *F*_(2,72)_ = 23.95, ****P < 0.0001; two-way ANOVA). Lower panel shows that MD induced a significant increase in climbing behavior and a significant decrease in immobility in the FST (Test, *n* = 7 in each group, climbing: *P < 0.05; immobility: *P < 0.05) in juvenile rats (P21–P28). Administration of ketamine (20 mg/kg) attenuated MD-induced changes in climbing and immobility 24 h post-injection (Test, climbing: *F*_(1,24)_ = 26.69, ****P < 0.0001; immobility: *F*_(1,24)_ = 18.76, **P < 0.01; two-way ANOVA). All data is represented as means ± SEM.

**Figure 2:**
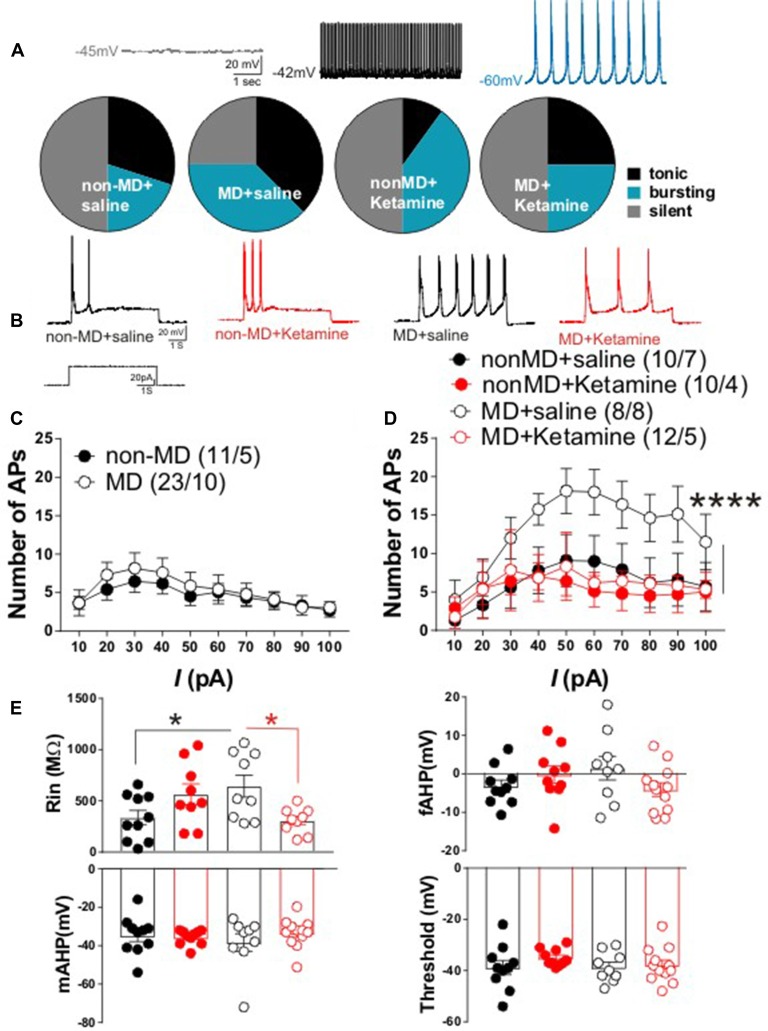
Ketamine normalized MD-induced changes in lateral habenula (LHb) intrinsic excitability and firing patterns in late-adolescent rats. **(A)** Distribution of LHb neurons based on firing patterns in whole cell current-clamp recordings of spontaneous activity. The insets show example traces from LHb neurons with silent, tonic and bursting patterns recorded from non-MD or MD rats that received either saline or ketamine injections (recorded 72 h post-injection). The number of cells/rats are similar to the ones reported in **(D)**. MD increased spontaneous LHb neuronal activity (specifically bursting) and ketamine reversed these effects of MD. **(B)** Representative traces from LHb neurons in non-MD and MD rats that received either saline or ketamine (20 mg/kg) injections and sacrificed after 72 h. **(C)** Figure shows the average of whole cell action potential (AP) recordings in response to depolarizing current injections (*I*) with intact synaptic transmission in LHb slices from P42 to P50 non-MD and MD rats. MD did not alter LHb neuronal excitability with intact synaptic transmission. **(D)** Whole cell patch clamp recordings of action potentials (APs) in response to depolarizing current injections *(I)* with blocked synaptic transmission from LHb neurons in non-MD and MD rats that received either saline or ketamine (20 mg/kg) injections and sacrificed after 72 h. MD increased LHb intrinsic excitability and ketamine reversed this effect (*F*_(3,360)_ = 15.84, ****P < 0.0001; two-way ANOVA). **(E)** Measurements of intrinsic properties of LHb neurons including input resistance (Rin), fast afterhyperpolarization (fAHP), medium afterhyperpolarization (mAHP) and AP threshold. Average amplitude of fAHP, mAHP and Rin were generated from recordings in **(D)**. MD significantly increased Rin and ketamine reversed this effect of MD (*F*_(1,33)_ = 3.936, *P < 0.05; two-way ANOVA). Numbers indicated in **(C,D)** represent the number of cells recorded per rats in each group.

### Electrophysiology

Whole cell recordings were performed on LHb slices using a patch amplifier (Multiclamp 700B) under infrared-differential interference contrast microscopy. Data acquisition and analysis were carried out using DigiData 1440A, pClamp 10 (Molecular Devices, Union City, CA, USA), Clampfit and Mini Analysis 6.0.3 (Synaptosoft Inc.). Signals were filtered at 3 kHz and digitized at 10 kHz. The recording ACSF was the same as the cutting solution except that it was ascorbic acid-free. Spontaneous LHb activity, firing patterns and resting membrane potentials (RMP) were assessed using current-clamp recordings of action potentials (APs) for 3 min with no injected current immediately after whole cell configuration where neurons remained at their own RMPs. LHb neurons were classified as silent, tonic or bursting based on spontaneous patterns of firing. Neuronal excitability recordings in response to injection of depolarizing currents were conducted as previously described (Authement et al., 2018). LHb neurons were given increasingly depolarizing current steps at +10 pA intervals ranging from +10 pA to +100 pA, allowing us to measure AP generation in response to membrane depolarization (5 s duration). Current injections were separated by a 20 s interstimulus interval and neurons were kept at −65 mV with manual direct current injection between pulses. Synaptic transmission blockade was achieved by adding 6,7-dinitroquinoxaline-2,3-dione (DNQX; 10 μM), picrotoxin (100 μM) and APV (50 μM) to block AMPA, GABA_A_ and NMDAR-mediated synaptic transmission, respectively. Synaptic blockers were present from the start of each recording. The number of APs induced by depolarization at each intensity was quantified and averaged for each experimental group. Measurements of AP threshold, medium afterhyperpolarization (mAHP) and fast afterhyperpolarization (fAHP) amplitudes, and input resistance (Rin) were conducted as described previously (Authement et al., 2018). In brief, AP threshold was measured at the beginning of the upward rise of the AP. mAHP was measured as the difference between AP threshold and the peak negative membrane potential at the end of the current step. fAHPs were calculated as the difference between AP threshold and the peak negative potential following the AP. Rin was determined by injecting a small (50 pA) hyperpolarizing current pulse (5 s) and calculated by dividing the steady-state voltage response by the current pulse amplitude. Measurements of AP characteristics were obtained using Clampfit.

### Animal Behavior

The modified rat FST was conducted as previously described (Detke et al., 1995). As a standard method, rats underwent two swim sessions in a glass cylinder (20 cm diameter × 30 cm depth) filled with water (25°C ± 1°C) as it has been shown that juvenile Sprague-Dawley rats usually adopt immobility as a passive coping strategy from P20 onwards and need two swim sessions to develop this coping strategy in the FST (Martínez-Mota et al., 2011). In the first session (Pre-test), animals were allowed to swim for a total of 15 min. Rats were then dried and returned to the home cage. Rats underwent the second swim session 24 h later, but for only 5 min (Test). With respect to experiments involving ketamine, animals were injected i.p. with either ketamine (20 mg/kg) or equivalent amount of vehicle (saline) 1 h after the first session and the second session was conducted 24 h later. We chose this dose because ketamine reliably reduced immobility in the FST at this dose in late adolescent/young adult rats (Parise et al., 2013). In both the pre-test and test, a time-sampling method was used to quantify three behavioral outputs: climbing, swimming, and immobility. Definitions of behavioral outputs were scored as previously described (Browne et al., 2015). For electrophysiological experiments with ketamine, we sacrificed the late-adolescent animals 72 h after a single ketamine administration to investigate the prolonged effects of ketamine on LHb neuronal excitability and firing pattern.

### Statistical Analysis

Values are presented as means ± SEM. Statistical significance was determined using unpaired Student’s *t*-test or two-way ANOVA with Bonferroni *post hoc* analysis with a significance level of *p* < 0.05. Statistical analysis of electrophysiological recordings was based on the number of recorded cells. In behavioral experiments, pre-planned Bonferroni multiple comparisons were conducted. All statistical analyses were performed using Origin 2016 or GraphPad Prism 7.

## Results

### Maternal Deprivation-Induced Behavioral Immobility Was Reversible by Ketamine

To test whether MD is associated with a depressive-like behavioral phenotype in late adolescence, we subjected non-MD and MD rats (P42–P50) to the FST. MD significantly increased immobility time while decreased time spent swimming or climbing in the FST, suggesting that MD reliably induces a depressive-like phenotype in late adolescence (Figure 1A, lower panel).

Interestingly, juvenile MD pups (P21–P28) exhibited increased climbing behavior with a significant decrease in immobility in the FST, compared to age-matched non-MD animals (Figure 1B, lower panel). To examine the protracted antidepressant effects of ketamine, we subjected rats (P42–P50) to the FST 24 h after a single *in vivo* administration of ketamine (20 mg/kg) or saline. Ketamine reduced the MD-induced increase in immobility and increased time spent swimming in late adolescent rats (Figure 1A, lower panel). Strikingly, ketamine was also able to normalize the MD-induced differences in behavior in the FST in juvenile rats (P21–P28). Ketamine increased immobility time while decreasing the exaggerated climbing behavior in MD rats compared to controls (Figure 1B, lower panel). Irrespective of the FST on the test day, both juvenile and late-adolescent non-MD and MD rats quickly adopted the passive coping behavior (immobility) in the first swim session (pre-test; Figures 1A,B, upper panels).

### MD-Induced Changes in LHb Spontaneous Activity and Intrinsic Excitability Were Normalized by Ketamine

LHb hyperactivity seems to be the common finding in rodent models of depression and in humans with depression (Sartorius et al., 2010; Li et al., 2011; Meye et al., 2015; Neumann et al., 2015; Lawson et al., 2017). Recently we demonstrated that in early-mid adolescent rats (P21–P28), MD dysregulates CRF signaling and leads to a downregulation of SK2 (a Ca^2+^ activated potassium channel) which contributed to LHb hyperexcitability (Authement et al., 2018). We tested whether MD-induced increases in LHb neuronal excitability persisted into late adolescence (P42–P50). Furthermore, we investigated whether the observed persistent antidepressant behavioral effects of ketamine (Figure 1A, lower panel, 24 h after ketamine administration) were associated with reversal of MD-induced changes in LHb neuronal excitability and firing patterns in late adolescent rats 72 h after ketamine administration. We found that the percentage of neurons which were spontaneously active (tonic or bursting) in current clamp recordings with intact synaptic transmission was larger in late adolescent MD rats compared to control non-MD rats. In addition, ketamine was able to normalize this effect of MD on LHb neuronal activity (Figure 2A).

In addition to current-clamp recordings of basal neuronal firing, we also tested whether LHb neurons from late adolescent MD rats exhibited enhanced firing in response to depolarization compared to control non-MD rats in the absence or presence of fast synaptic transmission. We did not detect a significant difference in LHb neuronal excitability between non-MD and MD rats in intact synaptic transmission in response to 10–100 pA depolarizing current steps (Figure 2C). MD significantly increased LHb intrinsic excitability in response to depolarization when fast synaptic transmission was blocked and ketamine robustly blocked this MD-induced increase in LHb intrinsic excitability 72 h post-injection (Figures 2B,D). Furthermore, the MD-induced increase in intrinsic excitability was associated with a significant increase in Rin (without any change in the amplitude of fAHP, mAHP or threshold) that was reversible by ketamine. This suggests possible decreases in potassium conductances underlying membrane resistance in LHb neurons that can also be targeted with ketamine (Figure 2E).

## Discussion

We have provided evidence for long-lasting antidepressant effects of a single *in vivo* administration of ketamine in reversal of LHb neuronal dysfunction (72 h post-injection) and depressive-like behavioral abnormalities (24 h post-injection) associated with a severe early life stress (MD). MD rodents and adolescent human subjects reporting low parental care, show heightened impulsivity and exhibit anxiety-and depression-like behaviors associated with altered monoaminergic neurotransmission (Heim et al., 2010). Consistently, we also observed that MD induces a pro-depressive behavioral phenotype in the FST in late adolescent rats. Interestingly, we found that juvenile rats showed an increased active coping behavior in the FST (with an increase in climbing behavior) which indicates a developmental shift in the behavior of MD animals in the FST. Ketamine was able to reverse abnormal behavioral phenotypes in mid and late adolescent rats. More importantly, we were able to demonstrate the ability of ketamine in persistently reversing MD-induced changes in LHb neuronal excitability and firing patterns in late-adolescent rats up to 72 h post-injection. MD was accompanied by an increase in LHb intrinsic excitability and enhanced bursting mode of LHb neuronal firing. These aberrant LHb neuronal activity were ameliorated with a single i.p. administration of ketamine 72 h post-injection. Consistent with our findings, a recent study demonstrated that ketamine blocks LHb neuronal bursting that underlies behavioral depression and anhedonia in two rodent animal models of depression through local NMDAR-dependent blockade in the LHb (Yang et al., 2018). However, the effects of ketamine in this study were evaluated only 4 h after injection. It is yet to be determined how long ketamine-induced suppression of LHb bursting activity persists in these depression models. Furthermore, it is unclear whether NMDAR antagonism underlies ketamine’s prolonged effects. In fact, non-NMDAR-mediated glutamatergic potentiation and sustained activation of AMPARs seem to be central to the protracted antidepressant effect of ketamine and its main metabolite, hydroxynorketamine (HNK; Moghaddam et al., 1997; Browne and Lucki, 2013; Zanos et al., 2016). It is also important to further investigate whether NMDAR- and non-NMDAR-dependent mechanisms of ketamine are involved in MD-induced LHb neuronal dysfunction and behavioral depression. Since LHb bursting involves both NMDARs and low-voltage-sensitive T-type calcium channels (T-VSCCs; Yang et al., 2018), possible developmental delays in NMDAR subunits/function (Roceri et al., 2002; Rodenas-Ruano et al., 2012) and/or T-VSCCs expression and function following MD could occur and contribute to the increase in LHb bursting activity following MD. However, given that ketamine only blocked MD effects on LHb neuronal excitability and firing patterns without affecting those of non-MD rats suggests that there might be an enhanced contribution of NMDAR after MD. Since MD-induced increase in LHb intrinsic excitability was accompanied by higher Rin, this also suggests possible decreases in potassium conductances underlying membrane resistance including leaky potassium currents that can also be targeted by ketamine. Given that brain-derived neurotrophic factor (BDNF) signaling is a key mediator of ketamine’s antidepressant properties (Browne and Lucki, 2013; Zanos and Gould, 2018) and that BDNF expression is altered following maternal separation or deprivation (Roceri et al., 2002; Shepard et al., 2018), possible BDNF-mediated alterations in potassium channel expression (Koo et al., 2012) could also be triggered by ketamine to normalize LHb neuronal excitability. In sum, our assessment of ketamine’s long-lasting behavioral and neurophysiological effects highlights the critical sensitivity of the LHb in MD-induced depression and subsequent responses to novel fast-acting antidepressants. This model should help to provide a framework for studying the currently limited treatment options available for adolescents suffering from major depressive disorder and higher risk of comorbidity later in life.

## Author Contributions

FN and RS designed the experiments and wrote the manuscript. RS, LL and FN performed electrophysiology experiments. RS and CB performed behavioral experiments. FN, RS, AB, CB and IL analyzed the data and prepared the figures.

## Conflict of Interest Statement

The authors declare that the research was conducted in the absence of any commercial or financial relationships that could be construed as a potential conflict of interest.

## Abbreviations

BDNF: brain-derived neurotrophic factor
DA: dopamine
fAHP: fast afterhyperpolarization
FST: forced swim test
LHb: lateral habenula
mAHP: medium afterhyperpolarization
MD: maternal deprivation
OFT: open field test
Rin: input resistance
T-VSCCs: low-voltage-sensitive T-type calcium channels
VTA: ventral tegmental area.
